# Inter‐ and intrasex habitat partitioning in the highly dimorphic southern elephant seal

**DOI:** 10.1002/ece3.7147

**Published:** 2021-01-29

**Authors:** Mark A. Hindell, Clive R. McMahon, Ian Jonsen, Robert Harcourt, Fernando Arce, Christophe Guinet

**Affiliations:** ^1^ Institute for Marine and Antarctic Studies University of Tasmania Hobart Tasmania Australia; ^2^ IMOS Animal Tagging, Sydney Institute of Marine Science Mosman New South Wales Australia; ^3^ Department of Biological Sciences Macquarie University North Ryde, Sydney New South Wales Australia; ^4^ Centre d’Etudes Biologiques de Chizé (CEBC) UMR 7372 Université de la Rochelle‐CNRS Villiers en Bois France

**Keywords:** Antarctic Shelf, foraging and diving behavior, Kerguelen Plateau, mid‐year haul out, predation risk, Southern Ocean

## Abstract

Partitioning resources is a key mechanism for avoiding intraspecific competition and maximizing individual energy gain. However, in sexually dimorphic species it is difficult to discern if partitioning is due to competition or the different resource needs of morphologically distinct individuals. In the highly dimorphic southern elephant seal, there are intersexual differences in habitat use; at Iles Kerguelen, males predominantly use shelf waters, while females use deeper oceanic waters. There are equally marked intrasexual differences, with some males using the nearby Kerguelen Plateau, and others using the much more distant Antarctic continental shelf (~2,000 km away). We used this combination of inter and intrasexual behavior to test two hypotheses regarding habitat partitioning in highly dimorphic species. (a) that intersexual differences in habitat use will not appear until the seals diverge in body size and (b) that some habitats have higher rates of energy return than others. In particular, that the Antarctic shelf would provide higher energy returns than the Kerguelen Shelf, to offset the greater cost of travel. We quantified the habitat use of 187 southern elephant seals (102 adult females and 85 subadult males). The seals in the two groups were the same size (~2.4 m) removing the confounding effect of body size. We found that the intersexual differences in habitat use existed before the divergence in body size. Also, we found that the amount of energy gained was the same in all of the major habitats. This suggests that the use of shelf habitats by males is innate, and a trade‐off between the need to access the large benthic prey available on shelf waters, against the higher risk of predation there. Intrasexual differences in habitat use are another trade‐off; although there are fewer predators on the Antarctic shelf, it is subject to considerable interannual fluctuations in sea‐ice extent. In contrast, the Kerguelen Plateau presents more consistent foraging opportunities, but contains higher levels of predation. Habitat partitioning in this highly dimorphic species is therefore the result of complex interplay of life history strategies, environmental conditions and predation pressure.

## Graphical abstract

Partitioning resources is key to avoiding intraspecific competition and maximizing individual energy gain. However, in sexually dimorphic species partitioning can be due to either competition or the different resource needs of morphologically distinct individuals. Our study demonstrates that habitat partitioning in highly dimorphic species is the result of complex interplay of life history strategies, environmental conditions, and predation pressure.

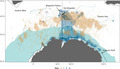

## 1 INTRODUCTION

How animals select feeding sites is a central theme in ecology because the quality of where animals feed has important implications for individual fitness and population viability (Bolnick et al., 2011). Animals must balance the costs of travel, intra and interspecific competition and predation risk against the energy gained. Individuals maximize fitness through individual specializations in feeding behavior (Dall et al., 2012) and/or by returning to sites that have consistently high productivity. Determining the trade‐offs between different foraging behaviors within a population is central to predicting how animals may respond to changes in their environment which is especially pertinent given continued, rapid climate change.

Maximizing energy gain may occur through habitat partitioning as this reduces intraspecific competition (Polis, 1984), especially in the presence of resource limitation and in highly variable, unpredictable and patchy environments (Bradshaw et al., 2004; Breed et al., 2011; Svanback & Bolnick, 2007). In highly sexual dimorphic species the sexes may differ in energetic, ecological or behavioral requirements leading to different patterns of resource use (Salton et al., 2019). This raises the question of whether different patterns of habitat use within a species are due to competition or the result of the intrinsically different resource requirements or foraging abilities.

Southern elephant seals (*Mirounga leonina*) are the most sexually dimorphic mammal species with adult males up to five times larger than adult females (Laws, 1953). At birth and for the first three years of life there is little sexual dimorphism, until males exhibit a postpubertal growth spurt at about 3–4 years (McLaren, 1993). Once fully grown, a small proportion of the largest males are responsible for siring most of the offspring (Le Boeuf et al., 2019) resulting in high variance in individual reproductive performance. Conversely, females maximize lifetime reproductive output by spreading reproductive effort over their lifetime, producing a single pup in the majority of years over their adult life (Desprez et al., 2018).

Southern elephant seals are distributed widely in the Southern Ocean (Hindell et al., 2016), and in the southern Indian Ocean they feed across 29 degrees of latitude (−40 to −69) and 150 degrees of longitude (0–150) a range that encompasses many habitat types of varying quality (Bailleul et al., 2007). Within this broad array of habitats, individual seals display a high degree of foraging site fidelity (Bradshaw et al., 2004), although how this develops and becomes fixed in the population remains unknown. What is known, is that foraging areas change as seals age (Authier et al., 2012; Chaigne et al., 2012; Field et al., 2005). Typically juvenile seals of both sexes forage in oceanic waters near their natal islands with no clear differences between males and females with respect to habitat, geographic location or foraging depths (McConnell et al., 1992), although they do show a bias to the east of their natal islands (Cox et al., 2020; McConnell et al., 2002; Orgeret et al., 2019). Post year one, their foraging ranges increase and they forage at deeper dive depths, reflecting enhanced diving abilities of the seals as they grow and mature (Hindell et al., 1999; Irvine et al., 2000). At 3–4 years, when growth rates diverge, females recruit into the breeding population while males continue to grow. At some point, foraging ranges also diverge, with males feeding predominantly on shelves and females in the open ocean (Bailleul et al., 2010; Martin et al., 2011). This pattern has been described for most populations of southern elephant seals (Campagna et al., 2000; Hindell et al., 2016, 2017) and is also seen in northern elephant seals (*Mirounga angustirostris)* (Le Boeuf et al., 2000; Robinson et al., 2012).

Another important factor in the distribution of animals in the Southern Ocean is the highly dynamic sea‐ice, which expands and contracts dramatically through the course of a year, and also with marked interannual variability (Simpkins et al., 2012). Sea‐ice plays a key ecological role in the Southern Ocean as a critical over‐winter nursery habitat for Antarctic krill and breeding substrate for several species of birds and mammals (Walther et al., 2002). For elephant seals, foraging success of adult females is negatively related to the extent of winter sea‐ice (McMahon et al., 2017) and in the southern Indian Ocean adult females avoid sea‐ice and move northwards as it grows (Labrousse et al., 2015). How males respond to changing sea‐ice has not been well documented, but some do remain in the ice for part of the winter (Bailleul et al., 2007).

However, habitat use is not attributable solely to sex, morphology or environmental factors. For example, female southern elephant seals from Iles Kerguelen use two distinct oceanic regions which results in differences in weaning mass of their offspring (Authier et al., 2012; Mestre et al., 2020). This has implications for population growth because weaning mass influences survival in elephant seals with larger weaners having higher survival than smaller conspecifics (McMahon et al., 2000; Oosthuizen et al., 2015) leading to higher rates of recruitment into the breeding population (Oosthuizen et al., 2018, 2019). For males, the two dominant habitats are different: the Kerguelen Plateau is adjacent to their haul out site at Iles Kerguelen and the animals can start foraging as soon as they leave the island, while the Antarctic continental shelf is over 1,500 km away and requires a journey of three weeks.

The pronounced inter and intrasex differences within this highly dimorphic species give rise to two hypotheses: (a) that males and females of the same size (for example adult females and subadult males) would use similar habitats, as there is no need for habitat partitioning until the sexes diverge in body size and exhibit differences in absolute energy requirements, and (b) that the Antarctic Shelf habitat will be a better foraging environment than the Kerguelen Plateau in terms of energy gain, in order to offset the added cost of travel. We therefore quantified four aspects of habitat use between similar sized adult female and male elephant seals: (a) broad‐scale habitat use (i.e., shelf versus oceanic waters), (b) seasonal changes in habitat use between the sexes, (c) habitat use and foraging behavior (dive depths and movement behavior) and (d) relative energy gain among habitats and sexes.

## 2 METHODS

We captured 102 adult female and 85 subadult male southern elephant seals at Iles Kerguelen between 2004 and 2019 at the end of their annual molt haul out. The seals were sedated (McMahon et al., 2000), weighed and measured (Field et al., 2002), and a CTD‐SRDL (Conductivity‐Temperature‐ Depth Satellite Relay Data Logger, Sea Mammal Research Unit, St Andrews, UK) attached to the hair on the seal's head (SMRU tag) (Field et al., 2012). The male elephant seals tracked from Iles Kerguelen since 2004 were subadults between 1.63 m and 3.18 m long, approximately 2–5 years of age (Carrick et al., 1962; Laws, 1953; McLaren, 1993). To control for the effect of body size we restricted our analyses to individuals of both sexes from the same size range. We defined this as the size distribution of the adult females, excluding the upper and lower one percentiles of this distribution to exclude outliers (Figure S1).

These tags provide: (a) regular location estimates, (b) summary dive profiles (duration, maximum depth and 4 time/depth inflection points for a random selection of all dives made (Fedak et al., 2002)) and (c) high resolution temperature and conductivity profiles, which were not used in this study. The weight of the tag and glue was 0.5 kg, or 0.15% of the mean departure weight. Previous studies have demonstrated that adult female seals carrying twice this load (instruments of up to 0.6% of their mass) were unaffected in either the short‐term (growth rates) or the long‐term (survival) (McMahon et al., 2008).

The at sea locations provided by Argos were filtered using a state‐space model via the R package *foieGras* (Jonsen & Patterson, 2020; Jonsen et al., 2020). We used a 24‐hr time step to provide a single location estimate per day to simplify the data set while still capturing the essential movement patterns of each individual.

We defined four general habitats, based on a priori knowledge of (a) the physical characteristics in each and (b) previous descriptions of habitat use in this population (Bailleul et al., 2010; Martin et al., 2011). We used GEBCO 14 bathymetric data to identify four habitats: The Antarctic continental shelf (0–2,000 m and south of 60°S), The Kerguelen Plateau (0–1,000 m and north of 60°S), The Indian Ocean East (>1,000 m and >70°E), and The Indian Ocean West (>1,000 m and <70°E). The Antarctic continental shelf habitat differs profoundly from the Kerguelen Plateau habitat in terms of bathymetry and ice cover, while the east and west oceanic regions differ in terms of eddy structure and productivity due to downstream iron transport and fertilization (Cotté et al., 2015; d'Ovidio et al., 2013; Green et al., 2020).

We also allocated each location as either; (a) in sea‐ice (sea‐ice concentration > 15% at that location on that day) or (b) not in sea‐ice (sea‐ice concentration ≤ 15%). We used daily sea‐ice maps from AMSR‐E & AVHRR (Parkinson & Cavalieri, 2012), accessed through the RAADTOOLS package in R (https://github.com/AustralianAntarcticDivision/raadtools).

### 2.1 Mid‐year haul outs

Mid‐year haul outs are a characteristic behavior of southern elephant seals, most common in juveniles of both sexes and in subadult males (Hindell & Burton, 1988; Orgeret et al., 2019). Given the location uncertainties, and that seals may haul out briefly just to rest, we defined a mid‐year haul out as when a seal was within 4 km of the coast for longer than 4 days after having been at sea for at least a month. As the Antarctic continental Shelf and the Kerguelen Plateau both extend for over one hundred kilometers no observations over the shelves are lost using this 4 km buffer. Most mid‐year haul outs occurred between April and May (Hindell & Burton, 1988), so we further restricted our analyses of haul out behavior to seals that were at sea for at least 150 days, to exclude seals with tags that failed early or which took some time to leave the island.

### 2.2 Individual habitat use

We determined the most frequently used of the four a priori habitats for each seal. Because habitat use varied over the postmolt period (Figure S2), we divided each postmolt migration into three stages. The timing of these stages varied both among (with timing of the male mid‐year haul out depending on their foraging location) and between the sexes (with most females not having a mid‐year haul out), so we defined these stages differently for males and females. For males, we defined three stages of 66 days each, excluding the first three weeks and the last three weeks of the foraging trips to allow for transit to foraging areas. For males, Stage 1 comprised days 21–87 of their first trip from Iles Kerguelen returning for the mid‐year haul out and the first postmolt stage. Stage 2 followed the mid‐year haul out, days 146–212, and Stage 3 days 213–279. Most females did not have a mid‐year haul out so we simply divided their time at sea into three 66 day stages, again excluding the first three weeks and the last three weeks of the trip to allow for transit time. The female stages were therefore defined as: Stage 1 days 21–87, Stage 2 days 88–154, Stage 3 days 155–221.

To account for changes in individual habitat use over time, we assigned the dominant individual habitat use for each winter stage. To do this we calculated the number of days that each seal spent in each of the four habitats during a stage and expressed this as a percentage. The habitat in which the seal spent the greatest percentage (McMahon et al., 2017) of its time was regarded as its *dominant habitat* for that stage.

### 2.3 Foraging behavior

We compared two metrics of foraging behavior among the sexes, habitats, and stage of the postmolt foraging trip. The first was based on move persistence values estimated from the state‐space models (Jonsen et al., 2018). These values range continuously from 0 to 1, with 0 indicating low levels of directional persistence suggesting Area Restricted Search and 1 indicating high directional persistence associated with rapid and directed travel.

The second foraging metric was designed to identify broad‐scale differences in prey. We did this by allocating each dive in the record as either benthic (maximum depth within 20 m of the bottom, based on GEBCO 14 bathymetric data) or pelagic (maximum depth further than 20 m for the ocean floor). We then calculated the percentage of dives transmitted each day that were benthic dives.

### 2.4 Estimating changes in body condition to indicate habitat quality

Body condition, which we use as a measure of habitat quality, was inferred from the drift rate of drift dives. Drift dives have a long, inactive phase during which the seal passively drifts in the water column, during which the rate and direction of the vertical displacement depends on the seals buoyancy (Arce et al., 2019a, 2019b). The buoyancy of seals each day determined by the ratio of lean tissue to blubber (Biuw et al., 2003), so negatively buoyant seals descend passively in the water column, while positively buoyant seals ascend. To estimate the mean daily drift rate for each seal we fitted a generalized additive model with a custom link function using the R package *slimmingDive* (Arce et al., 2019a, 2019b).

### 2.5 Statistical analyses

All results are reported as means ± standard error. All models were either linear mixed effects models (LMEs) or generalized linear mixed effects models (GLMEs) with individual seals as the random term. LME were used to test the size of seals and the relative move persistence relative to *sex*, *habitat* and *stage* of the year using the *nlme* package in R. All LMEs included an AR1 autocorrelation term to account for the serial dependence of daily tracking data. We used GLMEs with a binary error family to test the likelihood of being in sea‐ice or of making a benthic dive relative to *sex*, *habitat* and *stage* of the year using the *lme4* package in R. Given the large number of dives in the dataset (>1,000,000) we ran the models on a random subset of 100,000 dives. The models were fit using REML, and ranked by AICc, to ascertain the top models. Where several models were ranked highly (delta AICc < 2.0), the model with the fewest terms was taken to be the best under the rules of parsimony.

## 3 RESULTS

### 3.1 Overview of tracking data

We used a total of 187 seals (adult females = 102, males = 85 in the analysis. All seals were tagged at Iles Kerguelen between 2004 and 2019. The size distributions of each sex were similar (Figure S1); with a standard length of 2.33 ± 0.02 m for adult females and 2.37 ± 0.02 m for the subadult males (Table 1). For subadult males, the mean departure day of the year was 17 ± 2.6 (January 17). For females the mean departure day was several weeks later at 38 ± 2.4 (February 7).

**TABLE 1:** Details of the model selection for the analyses in the study. Only the top five models of full suite are presented, along with the null

	Model	Intercept	*df*	logLik	AICc	ΔAICc	weight
*a. standard length (lm)*							
1	Null	2.3	2	17.8	−31.6	0.0	0.5
3	*stl ~ sex*	2.4	3	18.5	−30.9	0.7	0.3
2	*stl ~ dominant habitat*	2.3	5	19.4	−28.5	3.1	0.1
4	*stl ~ dominant habitat + sex*	2.4	6	20.4	−28.3	3.4	0.1
8	*stl ~ dominant habtat + sex_dominant habitat:sex*	2.3	9	21.8	−24.5	7.1	0.0
*b. in sea‐ice (GLME ‐ binomial)*							
8	ice ~ sex + stage + sex*stage	−11.2	7	−6,470.1	12,954.2	0.0	1.0
4	*ice ~ sex + stage*	−10.2	5	−6,652.8	13,315.5	361.4	0.0
2	*ice ~ sex*	−10.8	4	−6,656.4	13,320.8	366.6	0.0
3	*ice ~ stage*	−9.3	3	−6,894.9	13,795.8	841.7	0.0
1	Null	−10.0	2	−6,898.3	13,800.7	846.5	0.0
*c. Move persistence (LME)*							
128	*Persistance ~ habitat + stage + sex +habitat:sex + habitat:stage + sex:stage + habitat:sex:stage*	0.5	27	15,793.6	−31,533.1	0.0	1.0
64	*Persistance ~ habitat + stage + sex +habitat:sex + habitat:stage + sex:stage*	0.5	21	15,702.3	−31,362.6	170.5	0.0
48	*Persistance ~ habitat + stage + sex +habitat:stage + sex:stage*	0.5	18	15,658.2	−31,280.3	252.8	0.0
56	*Persistance ~ habitat + stage + sex +habitat:sex + habitat:stage + sex:stage*	0.5	15	15,539.8	−31,049.6	483.5	0.0
32	*Persistance ~ habitat + stage + sex +habitat:sex + sex:stage*	0.5	19	15,524.1	−31,010.2	522.9	0.0
1	Null	0.6	4	13,350.3	−26,692.5	4,840.0	0.0
*d. Benthic diving (GLME – binomial)*							
128	*benthic ~ habitat + stage + sex +habitat:stage + habitat:sex + stage:sex + habitat:stage:sex*	−1.9	13	−19,285.9	38,597.7	0.0	1.0
48	*benthic ~ habitat + stage + sex +habitat:stage + stage:sex*	−1.9	10	−19,298.9	38,617.9	20.2	0.0
64	*benthic ~ habitat + stage + sex +habitat:stage + habitat:sex + stage:sex*	−1.8	11	−19,298.9	38,619.9	22.1	0.0
16	*benthic ~ habitat + stage + sex + habitat:stage*	−1.7	8	−19,302.4	38,620.8	23.0	0.0
32	*benthic ~ habitat + stage + sex +habitat:stage + habitat:sex*	−1.8	9	−19,302.3	38,622.7	24.9	0.0
1	Null	−1.3	2	−19,438.2	38,880.5	282.7	0.0
*e. Drift rate (LME)*							
39	*drift rate ~ stage + sex + stage:sex*	−0.2	9	21,245.6	−42,473.2	0.0	0.8
7	*drift rate ~ stage + sex*	−0.2	7	21,241.9	−42,469.8	3.4	0.2
48	*drift rate ~ habitat + stage + sex + habitat:stage + stage:sex*	−0.2	18	21,247.3	−42,458.5	14.8	0.0
3	*drift rate ~ stage*	−0.2	6	21,234.9	−42,457.8	15.4	0.0
16	*drift rate ~ habitat+stage + sex + habitat:stage*	−0.2	16	21,242.7	−42,453.4	19.9	0.0
1	Null	−0.2	4	21,207.7	−42,407.5	65.8	0.0

Tag performance varied considerably (Figure S3). For females, 60 out of 102 (59%) of the females had transmitting tags when they returned to Iles Kerguelen at the end of the postmolt trip (trip duration ~ 240 day). Tags on males did less well, with only 7 of 85 tags (8%) transmitting at the end of their trip (~300 day). The mean transmission duration for males was 211 ± 64 day and for females 206 ± 30 day. A full analysis of these data is available in Henderson et al. (2020).

**FIGURE 1:**
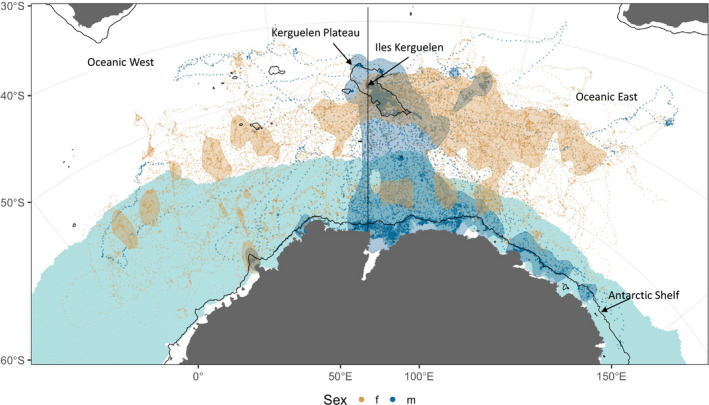
Map of the 30,151 daily location estimates from the State‐Space Models for postmolt elephant seals. Brown dots represent females and blue dots males. Also shown are the 50% kernel density isopleths for each sex. Black contour lines indicate the −1,000 m bathymetric contour and the vertical black line delineates the West and East Oceanic habitats. The blue shaded region represents the mean maximum ice extent for the years of the study

**FIGURE 2:**
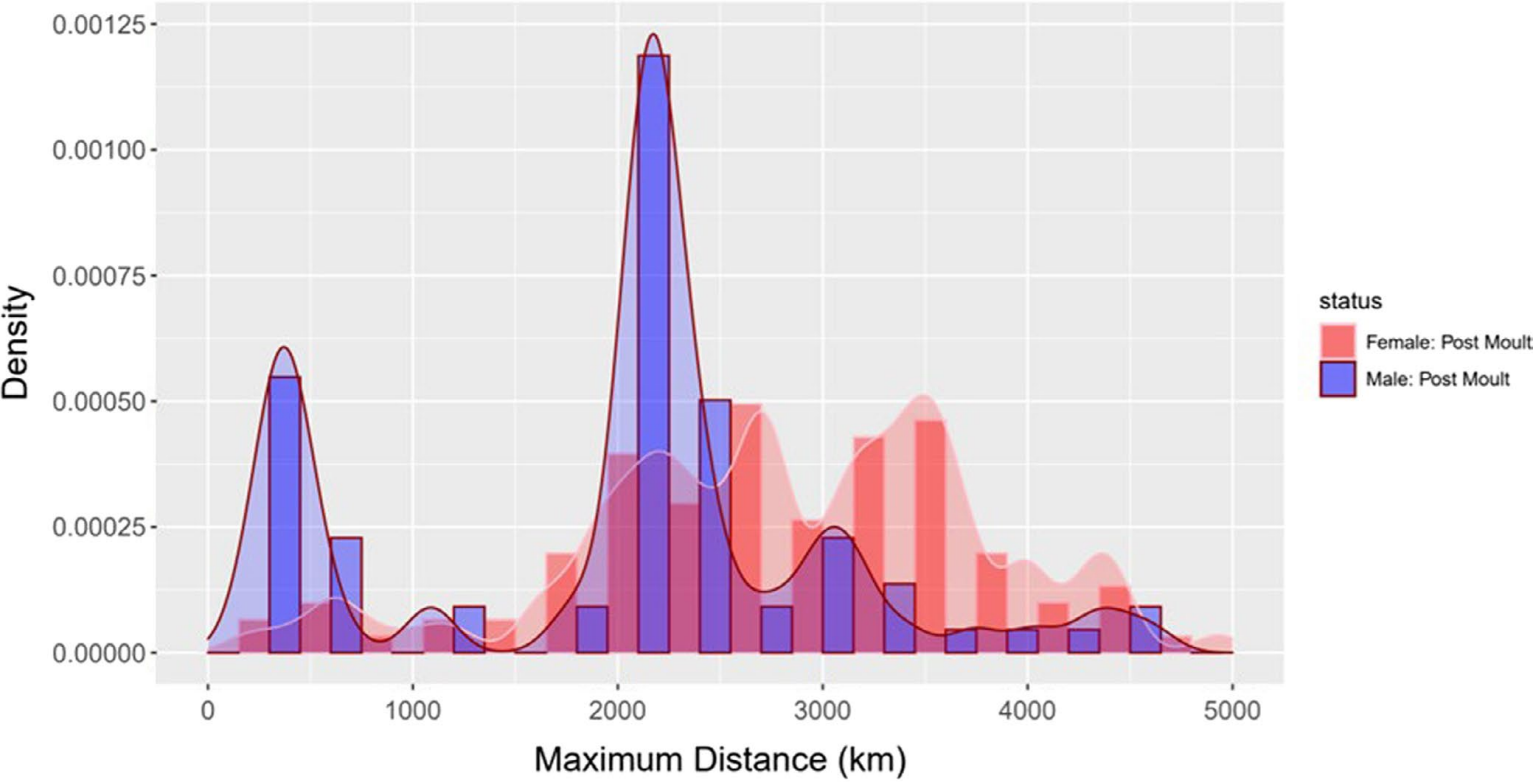
Frequency distribution on the maximum distance each seal went from Iles Kerguelen on its postmolt foraging trip

Overall, individuals of both sexes dispersed widely from Iles Kerguelen (Figure 1), primarily to the east, south and west. There were very few locations north of −45° (*n* = 681 out of 30,151 daily locations, or 1.6%). The females dispersed widely to the east and west of Iles Kerguelen (Figure 1) with a number (*n* = 18) briefly visiting the Antarctic Continental Shelf. The females travelled a median maximum distance of 2,791 km, with one travelling 4,931 km to the east of Iles Kerguelen (Figure 2). Subadult males also dispersed widely (Figure 1), but there was a concentration of locations on the Antarctic Continental Shelf and slope, as well as the Kerguelen Plateau. This was reflected in the bimodal distribution of distances travelled: one approximately 300 km from their departure point and the other 2,100 km away (Figure 2).

### 3.2 Mid‐year haul out

**FIGURE 3:**
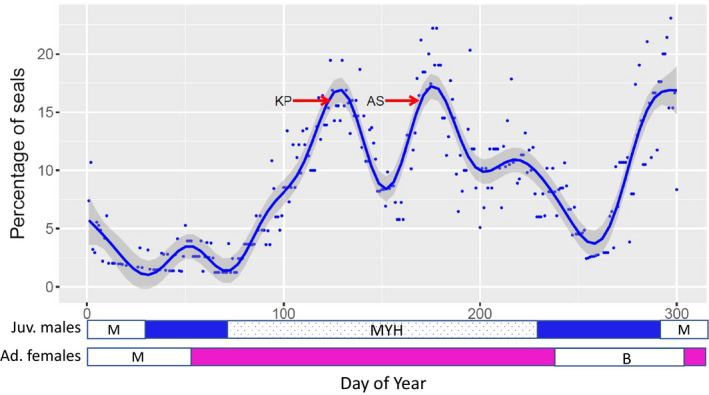
The percentage of postmolt males on land each day of the year. The solid line is a GAM smoother, with 95% confidence limits. The two arrows indicate the mean day of the start of the haul out for seals that predominantly used the Kerguelen Plateau (KP) and those that predominantly used the Antarctic Shelf (AS). Also shown, is a representation of the annual cycle of the juvenile males (blue) and adult females (pink). M indicates molting, B breeding and MYH the mid‐year haul out. Solid colors indicate when the majority of seals are at sea, and stippled indicates a period of asynchronous haul out

Only 9 (9%) of females at sea for at least 150 days made mid‐year haul outs, compared to 55 (83%) of the males. The percentage of males hauled out showed two distinct peaks 45 days apart (Figure 3), which may be related to the foraging locations of the individual seals. Seals that predominantly used the Kerguelen Plateau in the early part of the winter (see below) hauled out on day 123 (May 3), compared to seals that predominantly used the Antarctic Shelf, which hauled out on day 168 (June 17). Although 83% of all males did a mid‐year haul out, only 15%–20% were hauled out at any point, indicating that individuals haul out asynchronously. Low numbers of seals continued to haul out after July (~day 200).

### 3.3 Habitat Partitioning: intersex differences

**FIGURE 4:**
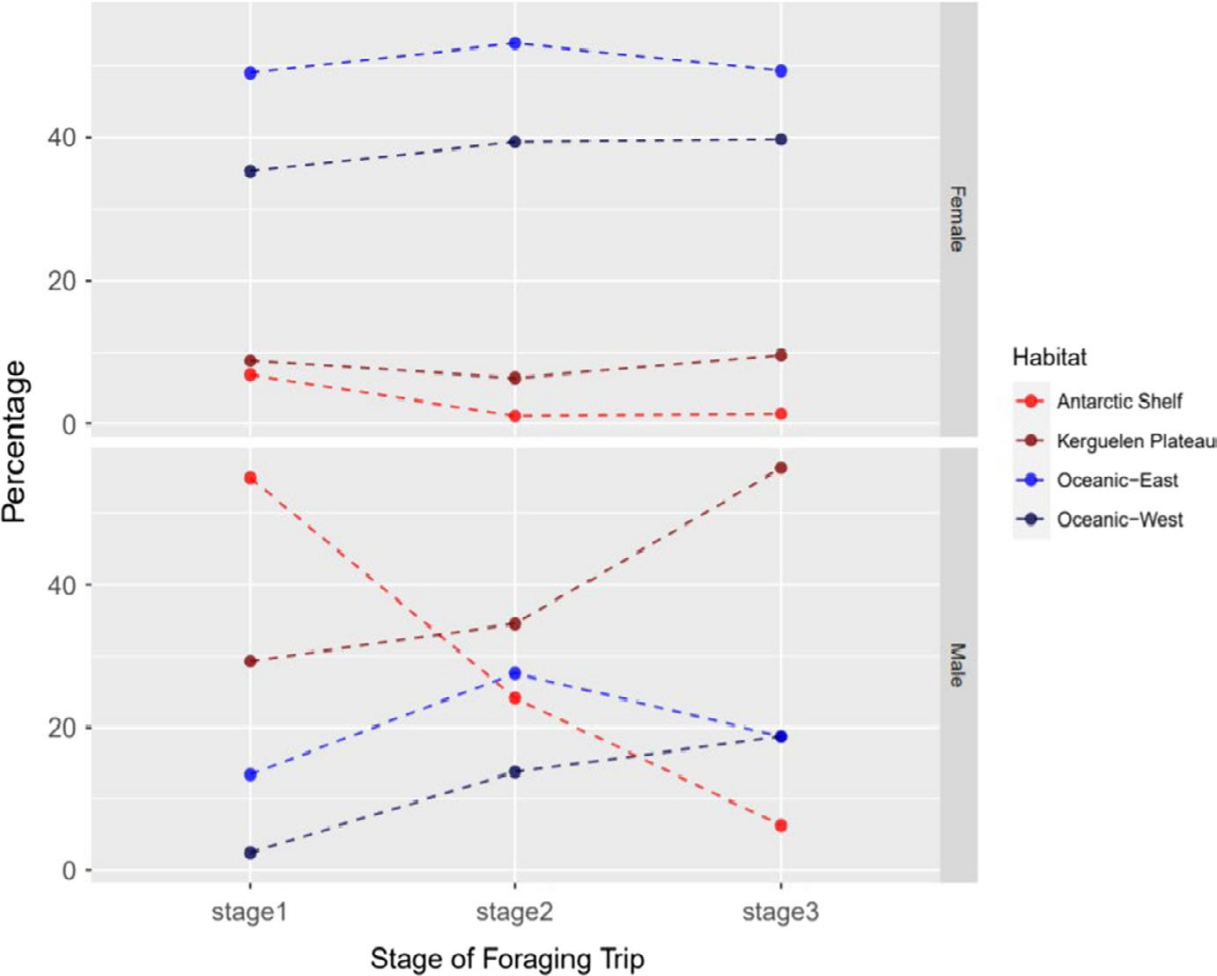
The percentage of seals that predominantly used of one of the four habitat types (Antarctic shelf, Kerguelen Plateau, Oceanic East and Oceanic West). Stage one (males and females day 21–88), Stage 2 (males day 117–183, females 88–154) and stage 3 (males day 213–279 and females day 155–221)

Sixty nine (84%) of subadult males predominantly used shelf regions (either Antarctic or Kerguelen Plateau) during *Stage 1* (Figure 4, Table S1). Females which, despite being the same body size as the subadult males, predominantly used oceanic regions (*n* = 86 (84%)). In *Stage 2* (after the mid‐year haul out), 17 of 29 males (58%) predominantly used shelf regions, compared to only 7 of 94 (7%) of females. By *Stage 3*, 10 of 16 (63%) of males were predominantly using shelf habitats, compared to just 8 out of 73 females (11%).

### 3.4 Habitat Partitioning: intrasex differences

Individuals of both sexes predominantly used one of the four habitats, the relative proportions of which varied during the postmolt foraging trip (Figure 4). Fifty five percent of the subadult males in *Stage 1* predominantly used the Antarctic Shelf and 29% used the Kerguelen Plateau. A further 13% used the Oceanic East habitat, while only 2% predominantly used the Oceanic West habitat. In *Stage 2* the percentage of males that used Antarctic Shelf dropped to 24% and those using the Kerguelen Plateau increased to 34%. By Stage 3, the percentage of males that used the Antarctic Shelf was only 6%, and the percentage using Kerguelen Plateau increased slightly to 56%. By that time the proportion of males using the Oceanic East and West Habitats was the same at 19% each. There was no difference in the size (standard length) of the seals with different patterns of habitat use (Table 1).

Fifty percent of postmolt females predominantly used the oceanic waters to the east of Iles Kerguelen, compared to 36% using the oceanic waters to the west. These proportions remained relatively consistent throughout the postmolt foraging period (Figure 4). The remaining 14% of the females predominantly used either of the shelf habitats, but unlike the males, there was little change over time.

### 3.5 Interactions with sea‐ice

**FIGURE 5:**
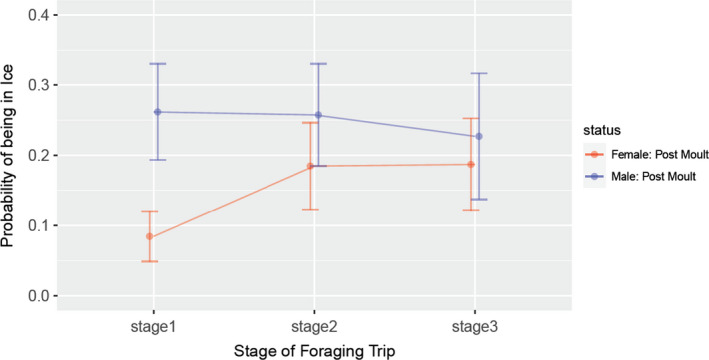
The probability of a location being in sea‐ice (> 15% concentration) for males and females throughout their postmolt foraging trip

A binomial GLME model indicated that males were much more likely to be located in sea‐ice than females (Table 1), especially in *Stage 1*, when this had a probability of 26% compared to only 8% for females (Figure 5). By *Stage 2*, the probability of males being in sea‐ice (but mostly not on the shelf – see above) was very similar (27%) while for females it increased from 8% to 18%. By *Stage 3* the likelihood of males being in ice decreased slightly to 22%, while females remained at 18%.

### 3.6 Foraging behavior in Southern Ocean habitats

**FIGURE 6:**
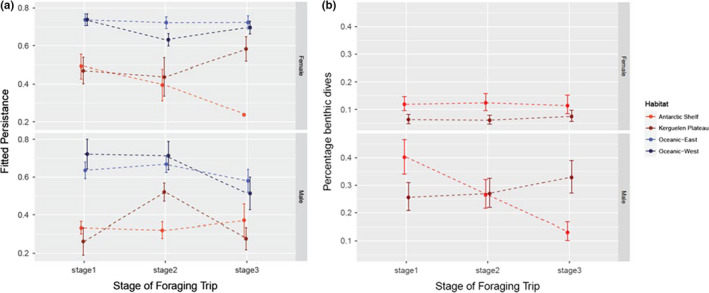
Changes in foraging behavior over time for seals in each habitat and each sex. (a) The fitted persistence values from a GLMM comparing each locations persistence value to the sex, habitat and stage of the postmolt foraging trip. (b) The mean percentage of benthic dives made by each sex in the two shelf habitats. Oceanic habitats were excluded as they were too deep for benthic dives

A LME model indicated that the spatial patterns of movement persistence varied among the habitats (Table 1). Overall, move persistence was lowest in the shelf habitats (Figure 6a), typically with values less than 0.5, indicating focal foraging behavior (Jonsen et al., 2020). The persistence values were much higher in the oceanic habitats with mean values ~0.8, indicative of more meandering travel. For the oceanic habitats this varied little among the stages for either sex (Figure 6a). For males, the animals on the Kerguelen Plateau had the lower persistence values during *Stages 1* and *3*, but this reversed in *Stage 2*. Females in the shelf habitats had higher persistence values than males in the same habitats, with the exception of those on the Antarctic Shelf in Stage 3, where they were similar to males (although this is based on a very small sample of females). The persistence values for males and females were similar in the oceanic environments, although in *Stage 3* males had lower persistence values.

The potential prey (pelagic versus benthic) of the seals varied among habitats and stages (Table 1), with benthic foraging more likely in shelf habitats for males than females (Figure 6b). Approximately 40% of dives made by males on the Antarctic Shelf were likely to be benthic in *Stage 1*, but this declined steadily throughout the winter to only 15%. The probability of males making benthic dives on the Kerguelen Plateau remained fairly constant (25%–35%) throughout the winter. Females were more likely to make benthic dives on the Antarctic Shelf (~10%) than on the Kerguelen Plateau (~5%).

### 3.7 Habitat quality: Relative foraging success

**FIGURE 7:**
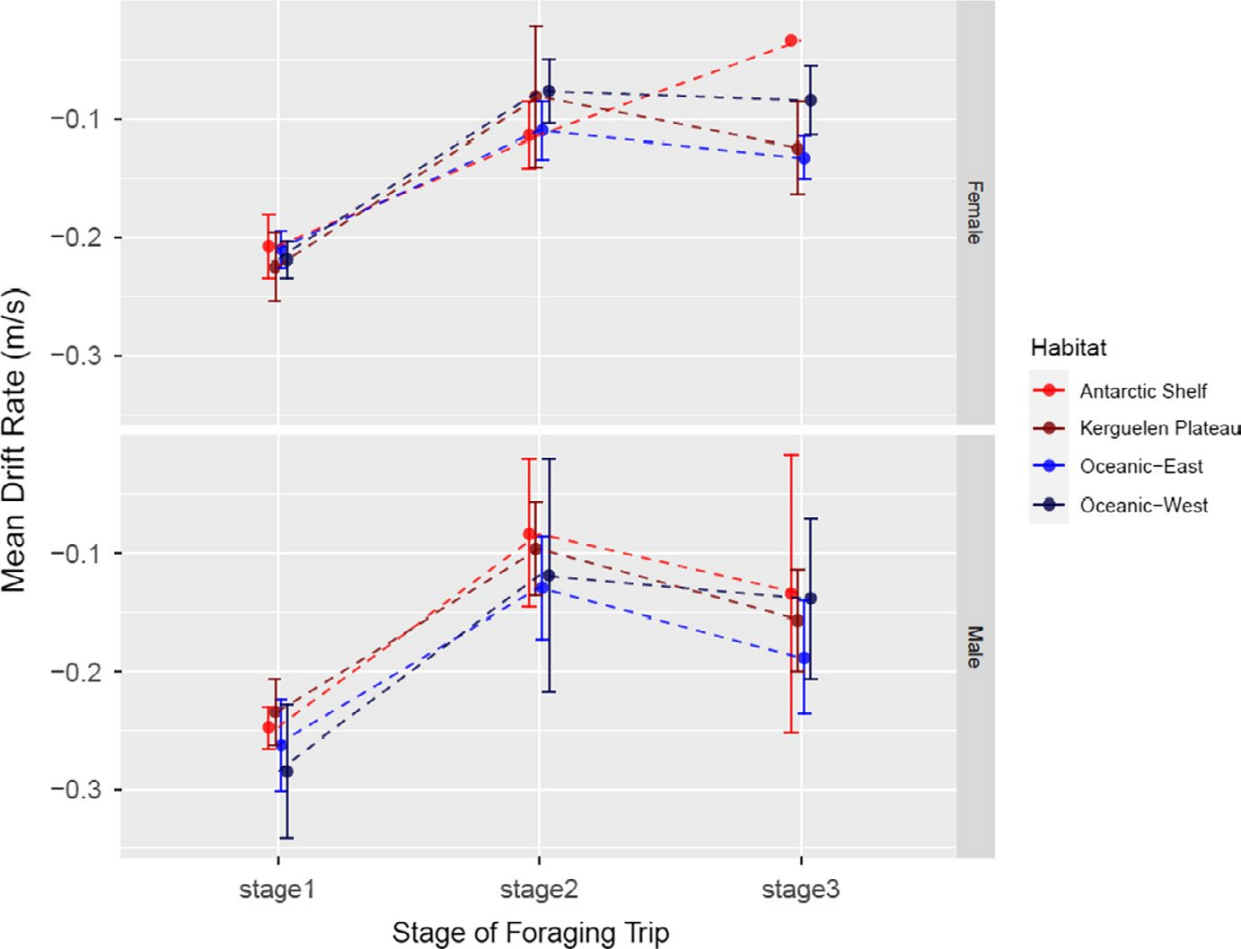
The mean daily drift rate (m/s) (and 95% confidence limits) throughout the postmolt foraging trip for each sex and habitat

A GLMM indicated that the best model describing drift rates (a proxy for habitat quality) included *sex* and *stage*, but not *habitat* (Table 1). Most change in body condition occurred during stage one (which excluded periods of transit), with little change after that (Figure 7). Females tended to be in poorer condition (i.e., lower drift rates) than males during *Stage 1* but, the sexes were equivalent in *Stages 2* and *3*.

## 4 DISCUSSION

Animals must successfully balance their energy needs across a suite of essential life activities including traveling, feeding and breeding. Those animals that balance their energy budget against costs such as predation most efficiently are the ones that have the highest breeding and survival prospects. Consequently, understanding the distribution of traits that maximize energy acquisition across a population and how each of these are related to animal performance is an essential component of quantifying the links between intraspecific variation in foraging traits (i.e., variability in foraging locations and fidelity to these areas). Here we described the inter and intrasex differences in habitat use in southern elephant seals. We found that similar sized male and female seals use different habitats, which is contrary to our hypothesis that habitat partitioning would not occur until males were larger than the females. We also found that the alternative habitats used within each sex provided similar foraging success, again in contrast to our hypothesis that the Antarctic shelf would be better foraging habitat for males.

Female seals rarely foraged in the Antarctic shelf regions, rather concentrating their feeding efforts in the mid‐ocean regions to the east or west of Iles Kerguelen. In contrast, subadult males focused their feeding on the Kerguelen Plateau or Antarctic Shelf regions. These differences are not simply due to age differences as adult male southern elephant seals retain this preference for shelf habitats (McIntyre et al., 2011; Pascoe et al., 2016). This has been attributed to the greater absolute energy requirements of adult males, which are five times bigger than adult females (Carrick et al., 1962; Laws, 1953). However, given that the animals in our study are comparable in size, and presumably have similar energy needs, there appears to be no energetic imperative for subadult males to use the shelf habitats, suggesting that this preference is an inherited behavior. While at sea metabolic requirements of elephant seals have rarely been quantified we assume it is similar for adult females and juvenile males. During the winter, adult females must recoup mass lost during molt as well as growing that year's pup. In contrast, males also have to recoup mass lost during molt and allocated energy to somatic growth. We assume that the energy for this for growth is roughly equivalent to the energy females require for gestation. Almost every other elephant seal population (northern and southern) displays similar habitat partitioning, despite very different breeding habitats, suggesting that it is an intrinsic property of the genus.

Southern Ocean shelves are home to large nototheniid species such as *Dissostichus* spp. and *Trematomus* spp. (De Broyer et al., 2014) which would provide greater return per unit effort than smaller meso‐pelagic prey such as squid and myctophids. A high proportion of dives made on the shelves by the males were benthic where they would have access to these larger species. In contrast, the few females that used shelf habitats tended to make pelagic rather than benthic dives, suggesting they exploit different prey. That males and females have different movement behaviors is further evidence that they exploit different prey types, and the more regionally focused ARS behavior of males is consistent with them feeding on relatively sedentary benthic prey. Why females do not use the same resources remains unknown but there may be other costs associated with feeding on the shelf, such as predation or interspecific competition.

Elephant seals have several predators, including killer whales (*Orca orcinus*) and sleeper sharks (*Somniosus antarcticus*) (van den Hoff & Morrice, 2008), which are concentrated in shelf habitats (Richard et al., 2017; Tixier et al., 2019). Using shelf habitats would therefore expose male elephant seals to greater predation risk (Bishop et al., 2020). Males have highly skewed reproductive outputs (i.e., only a very few males become beachmasters, but those that do sire a very large number of offspring (Le Boeuf, 1974)) and they may adopt the strategy of foraging on the shelf, despite higher predation risk, as it means they can attain larger body size rapidly and better compete for access to females on the breeding islands. Individuals of a number of species adopt riskier strategies, such as pursuing better quality food to improve reproductive potential (Clutton‐Brock & Isvaran, 2007; Engen & Stenseth, 1989; Owens, 2002). Females, on the other hand, have a much more uniform reproductive output restricted to a maximum of one pup per year, so the best way for them to maximize lifetime reproductive success is to live as long as possible (Desprez et al., 2018; Le Boeuf et al., 2019), and one way to do this is to reduce their exposure to predation risk (Gaynor et al., 2019).

Despite differences in habitat use between the sexes once they reach 3–5 years of age, there were also pronounced differences within each sex. Even though the majority of females used oceanic habitats, 30% of them predominantly used oceanic habitats to the west of Iles Kerguelen and 40% used waters to the east. The productivity of these two oceanic regions is controlled by contrasting oceanographic processes (d'Ovidio et al., 2013; d’ Ovidio et al., 2015) and have different distributions of meso‐pelagic prey due to the up and downstream effects of the Kerguelen Plateau; to the east, stronger eddy fields downstream of the plateau result in more patchy/concentrated prey than in the west where prey are more evenly distributed and also less abundant overall (Green et al., 2020). The different distribution and abundance of their primary food, meso‐pelagic fish (Banks et al., 2014; Cherel et al., 2008), may require different foraging strategies, that are learned during the early part of an individual's life and then retained for the life of the seal due to foraging site fidelity, (Authier, Bentaleb, et al., 2012; Bradshaw et al., 2004; Robinson et al., 2012).

Males also used two habitats, both of which were associated with shelf waters; the Kerguelen Plateau and the Antarctic Continental shelf which are very different in terms of the distances that the seals need to travel. Seals using the Kerguelen Plateau travel on average only 300 km, while those using the Antarctic Continental shelf must travel over 2,100 km, a journey of approximately three weeks which results in their mid‐year haul out being six weeks later than those using the Kerguelen Plateau. Despite this, there were no differences in energy gain between the two regions. Unexpectedly, the body condition of seals newly arrived on the Antarctic shelf after several weeks of travel was the same as those that did not have to travel at all and remained on the Kerguelen Plateau. This indicates that the Antarctic animals must have fed successfully during their transit, and so did not incur a cost associated with transiting. Documenting feeding during transit shows that feeding behavior, while concentrated in some areas, also occurs across a much wider area than previously thought. Our findings contradict those of Biuw et al. (2007) which indicated that body condition was relatively low on the Kerguelen Plateau. These differences could be due to (a) the relatively small sample sizes in that study, (b) the fact that males and females were not distinguished in the analysis, or (c) that foraging conditions have changed over the last two decades. Weaning mass of pups from females using sub‐Antarctic waters declined over the same time period, supporting the notion of declining foraging success in the southern Indian Ocean (Mestre et al., 2020).

There is also evidence that seals on the Kerguelen Plateau experience higher levels of predation than those on the Antarctic Continental Shelf. Satellite tag failures, a proxy for at sea mortality, were higher for males on the Kerguelen Plateau (Henderson et al., 2020) than on the Antarctic Shelf. Further, changes in isotopic signatures with age suggests mortality rates of young males are higher on the Kerguelen Plateau than on the Antarctic Continental Shelf (Chaigne et al., 2013). If the Antarctic Continental Shelf habitat has less predation than the Kerguelen Plateau seals should prefer to feed there, unless there is some other factor at play reducing its overall quality. This may be the presence of sea‐ice over the winter months which reduces access to the area (Hindell et al., 2017).

We hypothesize that these two male foraging strategies arise from a combination of learnt behavior and chance encounters with prey. Once males are large enough to feed benthically and can swim far enough to reach the Antarctic Shelf, (at 3–4 years of age) their inherent predisposition for shelf use becomes manifest. As they disperse southwards over the Kerguelen Plateau following the annual molt some will encounter rich patches of prey and stop, while others will not encounter rich patches and so continue moving south until they reach the Antarctic Shelf. This pattern appears to become fixed in subsequent years, as individuals return to these successful areas and foraging site fidelity becomes established (Martin et al., 2011), and the sooner they become faithful to a habitat the greater their likelihood of survival (Authier, Bentaleb, et al., 2012). Because Antarctic Shelf specialists can successfully forage on incidentally encountered prey *en route* (Thums et al., 2011) there are no additional energetic costs to the southern strategy. However, the encroaching sea‐ice limits how long they can use the Antarctic Shelf in the course of a year. Importantly, sea‐ice extent and concentration also varies considerably among years (Hindell et al., 2017) making it a more variable strategy in terms of long‐term energy gain. In contrast, the advantages of steady access to benthic habitats (and large prey) on the Kerguelen Plateau are offset by higher predation rates. The two strategies are therefore maintained in the population by the complex interplay of habit quality, predictability and risk.

The difference in both intra and interhabitat use in elephant seals has its origins in the highly dimorphic characteristics of southern elephant seals, and the imperative for males to attain a large body size to maximize individual reproductive output. Traits such as those linked to foraging locations can be fixed quickly in a population under changing environments in a polygynous system, because a few successful individuals contribute substantially more to future generations than less successful contemporaries (Loreau et al., 2019). This is especially true in elephant seals where only a few males (~4%), are responsible for most of the matings (Le Boeuf, 1974) and only a small proportion (~1% of all females born) of prime females contribute most to the next generation (Le Boeuf et al., 2019). Some experimental observations have shown that under extreme environmental change polygamous populations survive better than monogamous populations illustrating the importance of mating systems on population adaptive capacity and viability (Plesnar‐Bielak et al., 2012).

In elephant seals, the largest males usually have access to the most reproductively receptive females and they do this by defending harems while fasting for several months (Le Boeuf, 1972, 1974). This means that their success is related to their physical condition which is in turn due to foraging success. Consequently, we expect there to be strong selection for those traits that maximize size in male elephant seals, including a male's ability to identify those regions where high‐quality food is most abundant. There is a growing body of evidence linking environmental state, genotypes and phenotypes showing that the conditions animals are exposed to early in life can have profound transgenerational effects on performance and survival (Burton & Metcalfe, 2014; Donelson et al., 2018; Seebacher & Krause, 2019; Van Cann et al., 2019). This transgenerational plasticity may provide some buffering against the rapid climate changes that Earth is currently experiencing and allows animals time to respond (Donelson et al., 2018 and references therein).

## AUTHOR CONTRIBUTION

**Mark Hindell:** Conceptualization (equal); Data curation (equal); Formal analysis (equal); Writing‐original draft (equal); Writing‐review & editing (equal). **Clive R McMahon:** Conceptualization (equal); Data curation (equal); Formal analysis (equal); Funding acquisition (equal); Writing‐original draft (equal). **Ian Jonsen:** Data curation (equal); Formal analysis (equal); Methodology (equal). **Robert G Harcourt:** Conceptualization (equal); Writing‐original draft (equal). **Fernando Arce‐Gonzalez:** Formal analysis (equal); Methodology (equal); Software (equal); Writing‐original draft (equal). **Christophe Guinet:** Data curation (equal); Methodology (equal); Resources (equal); Writing‐original draft (equal).

## Supporting information

Figure S1.
Figure S2.
Figure S3.
Table S1.

## Data Availability

The seal data are available on the IMOS Australian Ocean Data Network (AODN) portal at: https://portal.aodn.org.au/search?uuid=06b09398‐d3d0‐47dc‐a54a‐a745319fbece.
