# Supplementary material for: Inter‐ and intrasex habitat partitioning in the highly dimorphic southern elephant seal
Source: Ecol Evol. 2021 Jan 29;11(4):1620–33. doi: 10.1002/ece3.7147 (PMC7882946; doi:10.1002/ece3.7147)
Supplement: Supplementary file 1 — Figure S1. Figure S2. Figure S3. Table S1. [file ECE3-11-1620-s001.docx]

Figure S1: Standard length (m) frequency distribution of southern elephant seals used in the study.


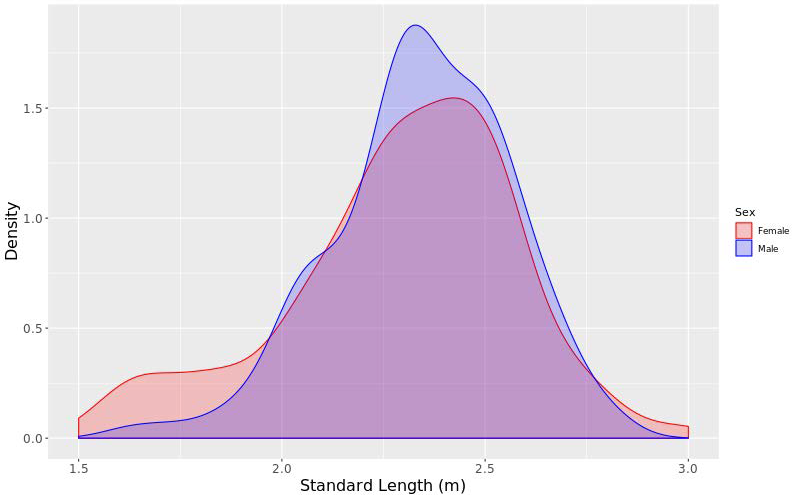


Figure S2 . *Map of the locations in each of three stages of the post-moult trip (see methods for definitions). Brown dots represent females and blue dots males. Also shown are the 50% kernel density isopleths for each group of seals. Black lines indicate the locations of the coastlines and the associated -1000m bathymetric contour. The blue shaded region represents the mean maximum ice extent for the years of the study*


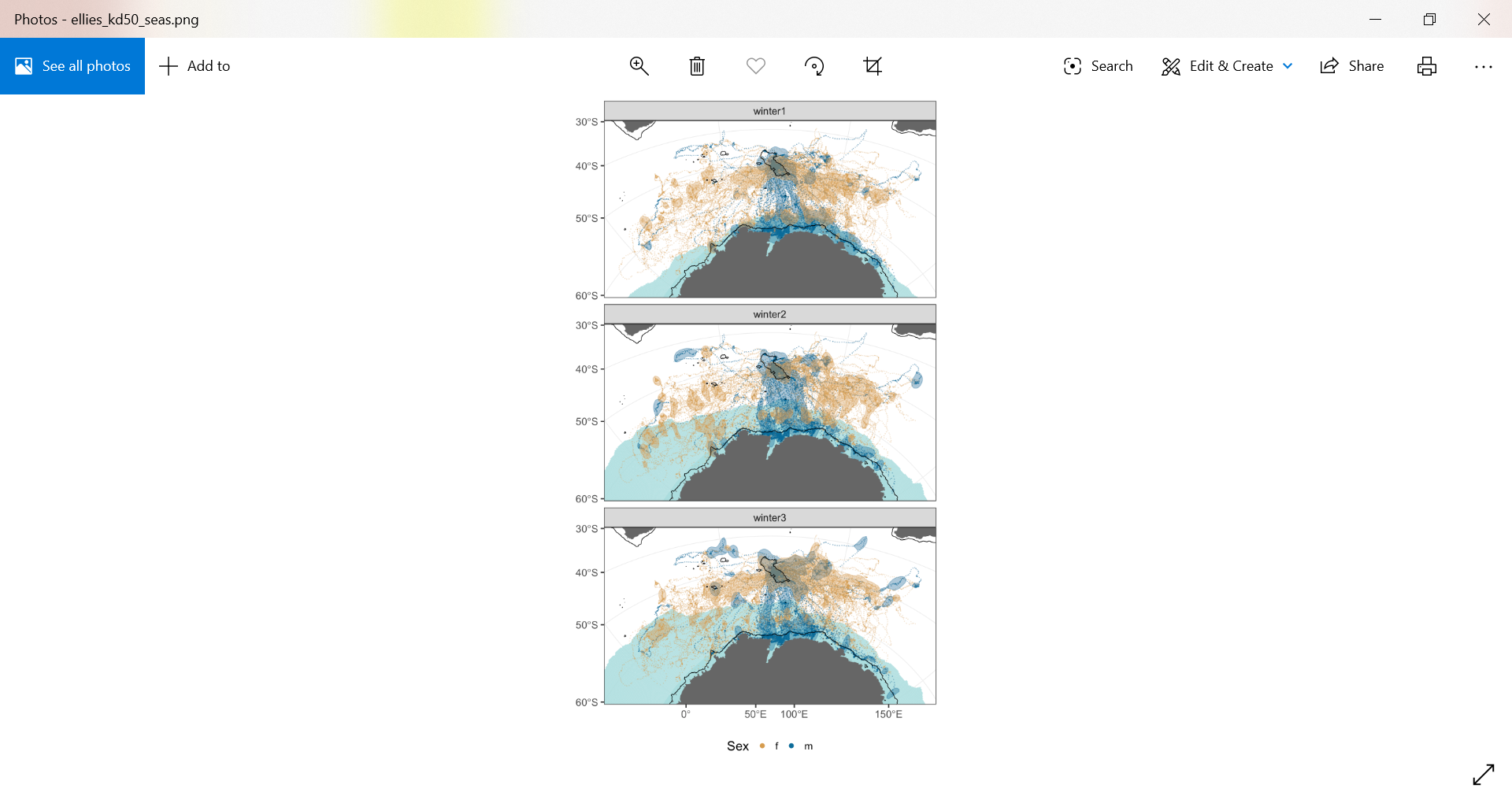


Figure S3: Tag performance expressed as the number tags transmitting (a) each day of the year and (b) each day after the seals departed Iles Kerguelen. Also indicated is the median return day of the trip for females (day 240), and the approximate return day of the males.


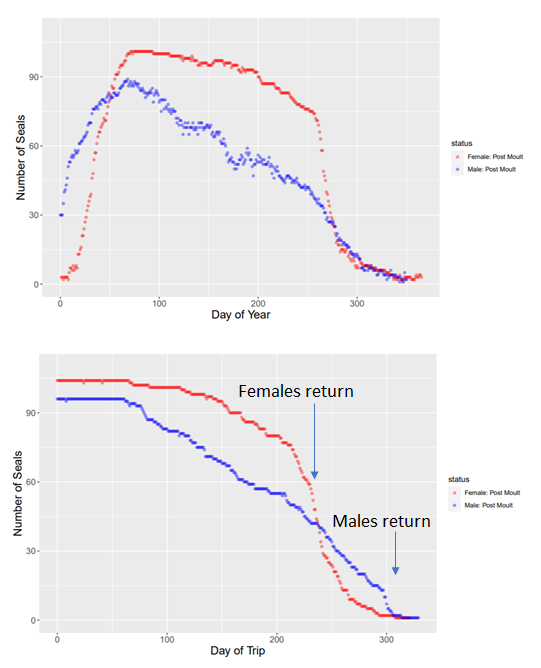


table S1: Table summary of the percentage of time that seals in each speciality spent in each habitat. An individual’s specialisation is defined as the habitat in which it spent the greatest amount of time during a particular stage of the post-moult trip.

|  |  |  | Females |  |  | Males |  |  |
| --- | --- | --- | --- | --- | --- | --- | --- | --- |
| stage | Specialisation | Habitat | N | % | sd | N | % | sd |
| stage1 | antshelf | antshelf | **7** | **65.8** | **16.6** | **45** | **88.1** | **13.8** |
|  |  | ocean_e | 3 | 17.2 | 11.0 | 27 | 15.0 | 11.7 |
|  |  | ocean_w | 6 | 30.8 | 12.9 | 10 | 6.8 | 3.9 |
|  |  | subshelf | 1 | 3.0 | - | 5 | 13.0 | 15.6 |
|  | ocean_e | antshelf | 4 | 11.0 | 17.9 | 5 | 28.5 | 14.3 |
|  |  | ocean_e | **50** | **95.4** | **10.3** | **11** | **83.2** | **18.3** |
|  |  | ocean_w | 2 | 6.1 | 2.1 | 1 | 15.2 | - |
|  |  | subshelf | 13 | 13.5 | 14.1 | 4 | 6.8 | 5.6 |
|  | ocean_w | antshelf | 4 | 15.2 | 14.9 | 1 | 3.0 | - |
|  |  | ocean_e | 2 | 3.8 | 1.1 | 0 | 0.0 | 0.0 |
|  |  | ocean_w | **36** | **94.4** | **11.7** | **2** | **98.5** | **2.1** |
|  |  | subshelf | 11 | 12.1 | 16.5 | 0 | 0.0 | 0.0 |
|  | subshelf | antshelf | 0 | 0.0 | 0.0 | 0 | 0.0 | 0.0 |
|  |  | ocean_e | 2 | 2.3 | 1.1 | 2 | 1.5 | 0.0 |
|  |  | ocean_w | 1 | 34.8 | - | 0 | 0.0 | 0.0 |
|  |  | subshelf | **9** | **95.6** | **12.0** | **24** | **99.9** | **0.4** |
| stage2 | antshelf | antshelf | **1** | **83.3** | **-** | **7** | **75.5** | **25.0** |
|  |  | ocean_e | 0 | 0.0 | 0.0 | 3 | 36.9 | 11.2 |
|  |  | ocean_w | 1 | 16.7 | - | 1 | 42.4 | - |
|  |  | subshelf | 0 | 0.0 | 0.0 | 1 | 18.2 | - |
|  | ocean_e | antshelf | 1 | 3.0 | - | 6 | 24.3 | 20.0 |
|  |  | ocean_e | **50** | **94.4** | **12.3** | **8** | **73.7** | **21.7** |
|  |  | ocean_w | 5 | 20.9 | 17.3 | 0 | 0.0 | 0.0 |
|  |  | subshelf | 9 | 19.3 | 12.0 | 4 | 16.0 | 7.1 |
|  | ocean_w | antshelf | 5 | 12.4 | 11.8 | 2 | 18.5 | 3.8 |
|  |  | ocean_e | 0 | 0.0 | 0.0 | 1 | 7.9 | - |
|  |  | ocean_w | **37** | **96.7** | **8.8** | **4** | **65.8** | **24.2** |
|  |  | subshelf | 4 | 15.2 | 17.5 | 3 | 30.6 | 3.6 |
|  | subshelf | antshelf | 0 | 0.0 | 0.0 | 1 | 19.4 | - |
|  |  | ocean_e | 1 | 34.8 | - | 4 | 12.3 | 14.0 |
|  |  | ocean_w | 1 | 48.5 | - | 1 | 38.7 | - |
|  |  | subshelf | **6** | **86.1** | **21.9** | **10** | **89.3** | **20.0** |
| stage3 | antshelf | antshelf | **1** | **57.6** | **-** | **1** | **100.0** | **-** |
|  |  | ocean_e | 0 | 0.0 | 0.0 | 0 | 0.0 | 0.0 |
|  |  | ocean_w | 1 | 42.4 | - | 0 | 0.0 | 0.0 |
|  |  | subshelf | 0 | 0.0 | 0.0 | 0 | 0.0 | 0.0 |
|  | ocean_e | antshelf | 0 | 0.0 | 0.0 | 0 | 0.0 | 0.0 |
|  |  | ocean_e | **36** | **97.5** | **4.4** | **3** | **96.4** | **3.8** |
|  |  | ocean_w | 1 | 6.6 | - | 0 | 0.0 | 0.0 |
|  |  | subshelf | 11 | 7.6 | 3.6 | 2 | 5.5 | 3.0 |
|  | ocean_w | antshelf | 0 | 0.0 | 0.0 | 1 | 10.6 | - |
|  |  | ocean_e | 2 | 4.5 | 0.0 | 2 | 6.1 | 2.1 |
|  |  | ocean_w | **29** | **96.0** | **6.8** | **3** | **89.9** | **9.3** |
|  |  | subshelf | 12 | 8.8 | 8.1 | 1 | 7.6 | - |
|  | subshelf | antshelf | 0 | 0.0 | 0.0 | 0 | 0.0 | 0.0 |
|  |  | ocean_e | 3 | 22.6 | 15.4 | 4 | 19.3 | 16.0 |
|  |  | ocean_w | 2 | 19.2 | 14.2 | 0 | 0.0 | 0.0 |
|  |  | subshelf | **7** | **84.8** | **15.8** | **9** | **91.4** | **14.1** |
